# Supplementary material for: Reconciling shared versus context-specific information in a neural network model of latent causes
Source: Sci Rep. 2024 Jul 22;14:16782. doi: 10.1038/s41598-024-64272-5 (PMC11263346; doi:10.1038/s41598-024-64272-5)
Supplement: Supplementary file 1 — Supplementary Information. [file 41598_2024_64272_MOESM1_ESM.docx]

## Supplementary information

### Supplement 1

**Inter-context correlation as a function of the dimensionality of the random context vector**

We used random vectors to index different latent causes to ensure that the representations for different latent causes would be approximately orthogonal. This idea is based on the fact that high dimensional random vectors, sampled from Gaussian distribution, are close to orthogonal. Here, we measure how orthogonal random vectors are as a function of dimensionality and the number of vectors (i.e., the number of latent causes).

Through a simple simulation, we found that – as the dimension increases – the values of pairwise correlation rapidly concentrate around zero (Figure S1a). Additionally, as the number of random vectors increases, the maximum pairwise correlation across any two vectors gets higher (Figure S1b), though the mean is relatively stable (Figure S1c).


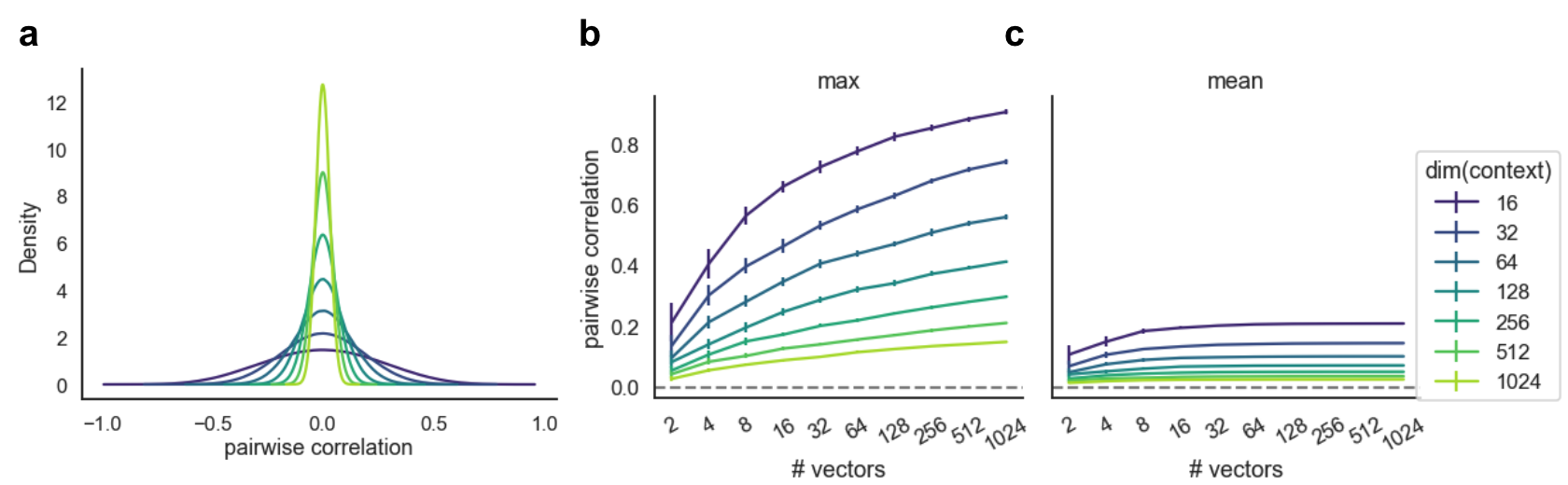


##### Figure S1

Statistics of pairwise correlation of random vectors as a function of vector dimension and the number of samples/vectors.

a) As the dimension increases, random vectors are more orthogonal.

b) As the number of vectors increases, the maximal pairwise correlation asymptotes. This upper bound is lower in higher dimensional space.

c) As the number of vectors increases, the mean pairwise correlation is relatively stable. Again, in high dimensional space, most vectors are close to being orthogonal. Error bar = 2SE across 50 simulations.

### Supplement 2

**In the interleaved condition in Simulation 2, some models “over-split” and some models “under-split.”**


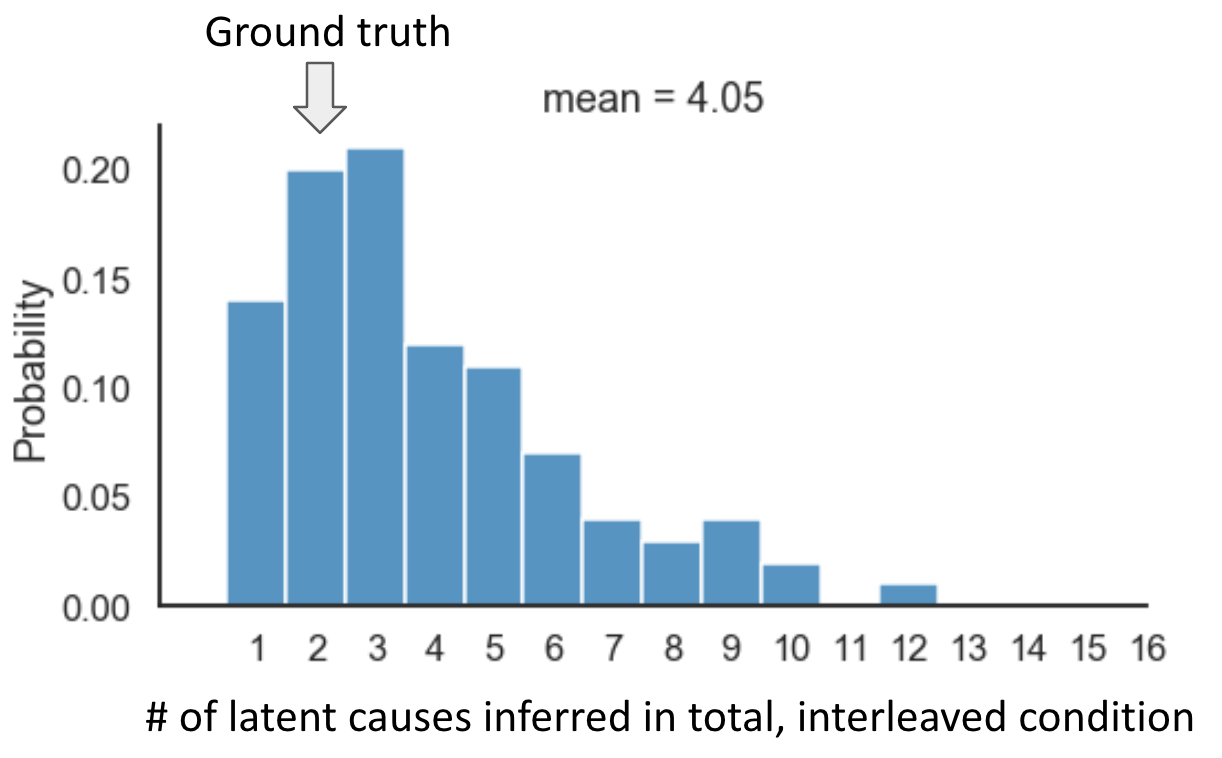


##### Figure S2

The distribution of the total number of latent causes inferred in the interleaved condition across (*N* = 100) models. Some models “over-split” (inferred more than two latent causes), and some models “under-split” (inferred only one latent cause). The main issue with models in the interleaved condition is that the correspondence between the inferred latent causes versus the ground truth latent causes was highly inaccurate (Figure 3h and i).

###

###

### Supplement 3

**The effect of the stickiness parameter on task performance in Simulation 2**

###
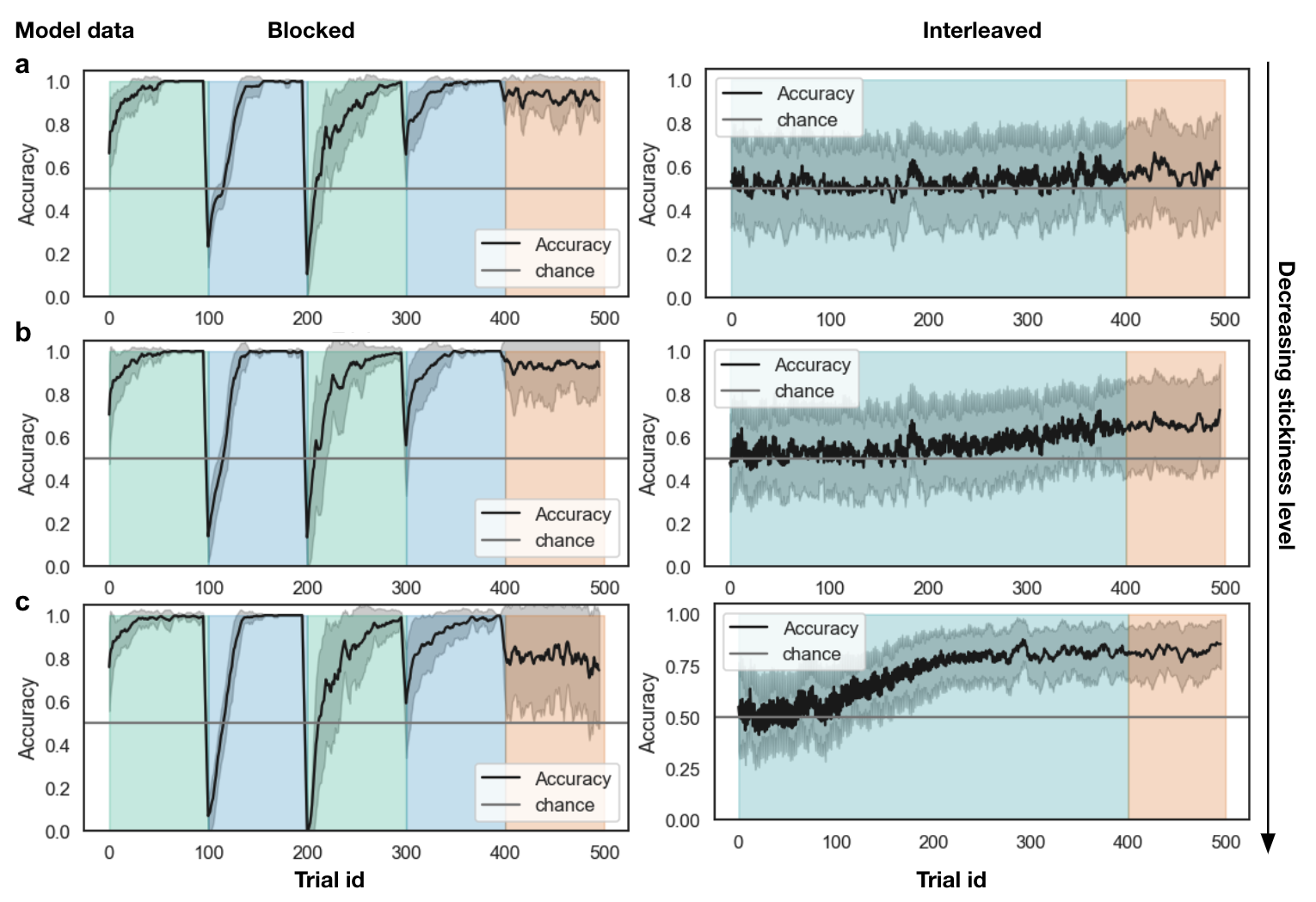


##### Figure S3

As the stickiness parameter decreases from 32 (a) to 8 (b) and then to 0 (c), the performance of LCNet during the test phase i) increases in the interleaved condition and ii) decreases in the blocked condition. This shows that the relative advantage of blocked over interleaved learning is a function of how well the model’s prior on temporal auto-correlation in the environment matches with the ground truth. Note that panel a is the same as Figure 3f and 3g in the main text. Error bands indicate 3SE. *N* = 20 models per condition.

###

###

### Supplement 4

#### **Episodic memory as a shortcut to full latent cause inference**

Full LCI in both SEM and the standard LCNet involves evaluating the posterior over all LCs, which is computationally expensive. We hypothesized that humans can leverage episodic memory to economize on LCI, by recalling the LC that was inferred previously for a particular sensory observation. Here, we present a proof-of-concept simulation using the task in Simulation 2 as a testbed.

Concretely, we equipped LCNet with an episodic memory buffer that maps observations to LCs (Figure S4a); this buffer acts as an efficient “shortcut” to the laborious full inference process, similar to the idea of amortization [^1^](https://paperpile.com/c/c9vAlV/6PmPY). The episodic memory mechanism stores previously encountered {observation, inferred LC} pairs. This episodic memory “shortcut” is turned on when its predictions become consistent with full inferences; when prediction error is too high, full inference is turned on and episodic memory is turned off. For additional detail, see the next section (“the implementation of episodic memory”).

Results from Simulation 2 show that models with episodic memory also qualitatively capture the human behavioral results – during the test phase, prediction performance was much better in the blocked condition compared to the interleaved condition (Figure S4b, c). Again, the reason is that LCI accuracy was much lower in the interleaved condition (Figure S4d, e). More importantly, we found that LCNet with episodic memory can save 96.20% of full inferences (Figure S5a, b) while still capturing the human data. With episodic memory, full inferences selectively happen at the first two block switches. Then episodic memory is sufficient for retrieving the proper LC for the rest of the task.

###
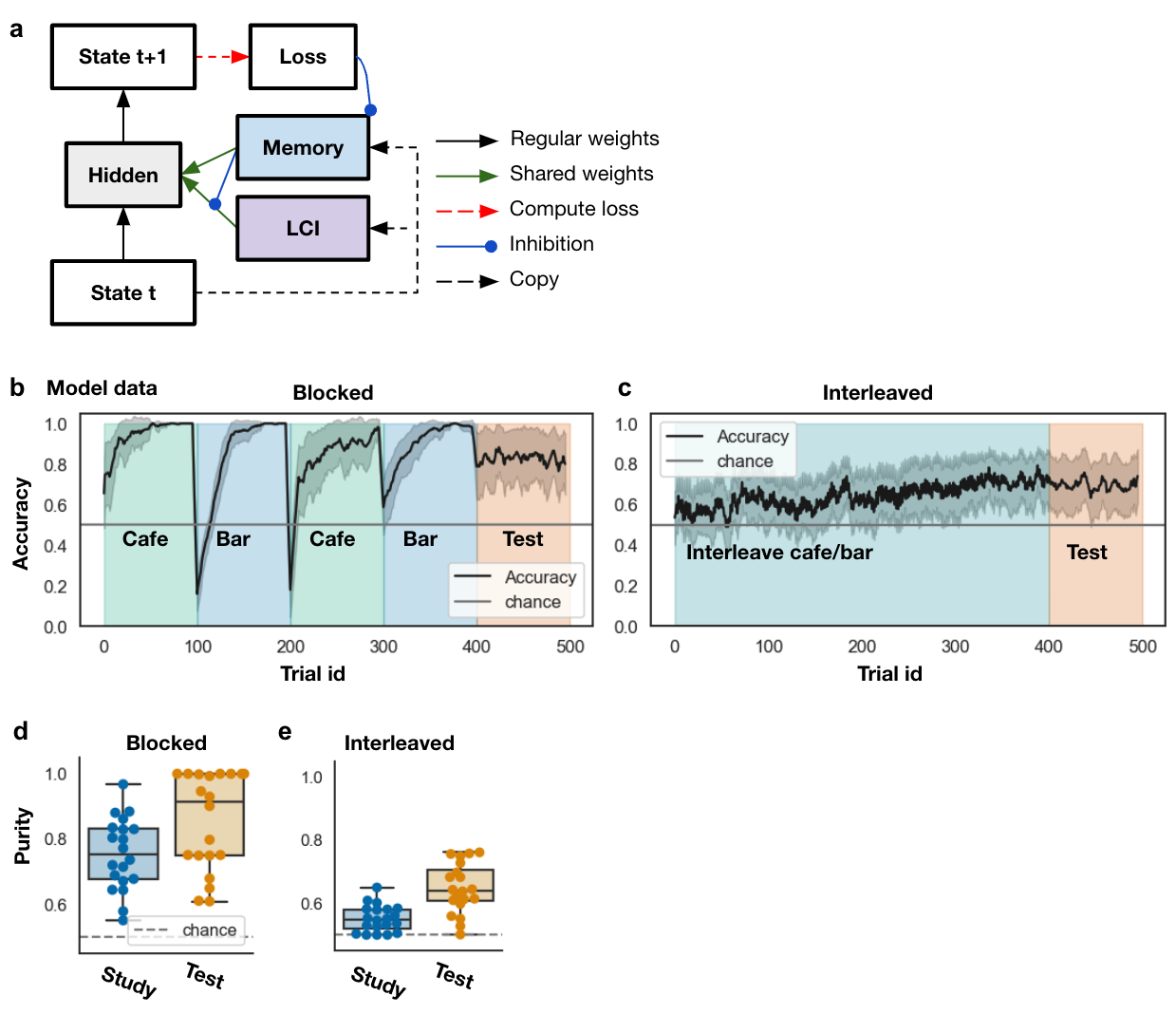


##### Figure S4

a) The model with the episodic memory mechanism. When the model retrieves a previously inferred latent cause from episodic memory, full LCI is suppressed. When the model uses retrieved LC and the current loss is too high, the model suppresses episodic retrieval and switches back to relying on full latent cause inference. “LCI” = latent cause inference.

b) and c) show the model performance over trials in the blocked vs. interleaved condition.

d) and e) show LCI cluster purity during the test phase in the blocked vs. interleaved condition for the model with episodic memory.

#####
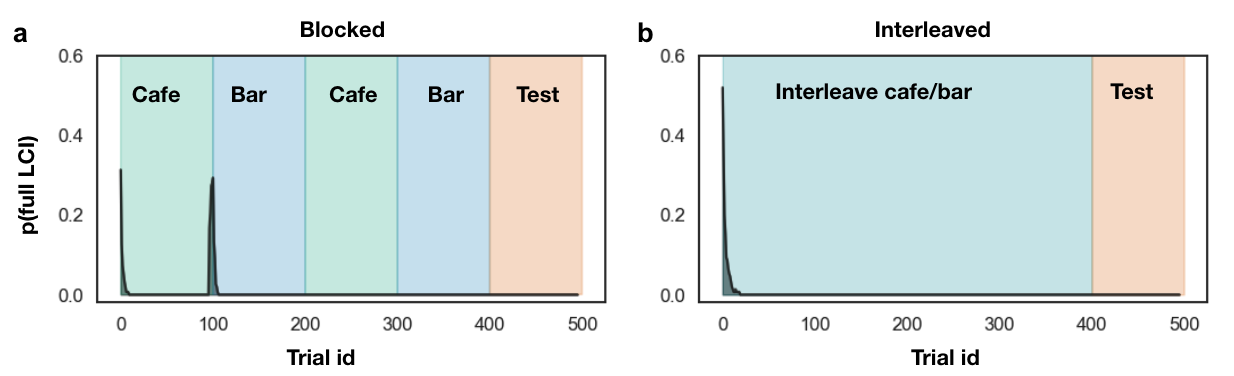


##### Figure S5

A large amount (96.20%) of full inferences can be saved with an episodic memory buffer. In the blocked condition (a), full inference mainly occurs at the first two event boundaries. In the interleaved condition (b), full inference mainly peaked at the beginning of the experiments.

*N* = 20 models per condition

####

#### The implementation of episodic memory

Episodic memory is implemented as a key-value dictionary, a simple proxy of content-based associative memory used for a wide range of cognitive tasks[^2,3^](https://paperpile.com/c/c9vAlV/TQUGn+LwTd2). In this framework, the key is used for memory search, and the value is used to store the content. To encode a new memory, the model stores the inferred LC (from the full inference procedure) as the value, paired with the current observation as the key. To retrieve a memory, it performs a one-nearest-neighbor (1NN) lookup[^2,4^](https://paperpile.com/c/c9vAlV/TQUGn+DYKRk) using the current observation as the key.

To ensure that outdated information does not lead to interference[^5,6^](https://paperpile.com/c/c9vAlV/4UPRi+m9GMm), we implemented a simple form of forgetting – the buffer is a queue that only keeps the most recent *M* (= 2) memories for each unique observation. M is chosen to be the smallest possible number that does not negatively affect model performance. To incorporate the assumption that the hippocampus has a narrow generalization gradient[^7–13^](https://paperpile.com/c/c9vAlV/D8dFG+Oq6ly+DWzYT+DtcZy+ofWCP+CrTZe+aZRDx), retrieval was designed to be conservative – given an observation *x*, it will return a LC if and only if there are at least *M* entries (LCs) associated with x, and they are all the same (Figure S5).


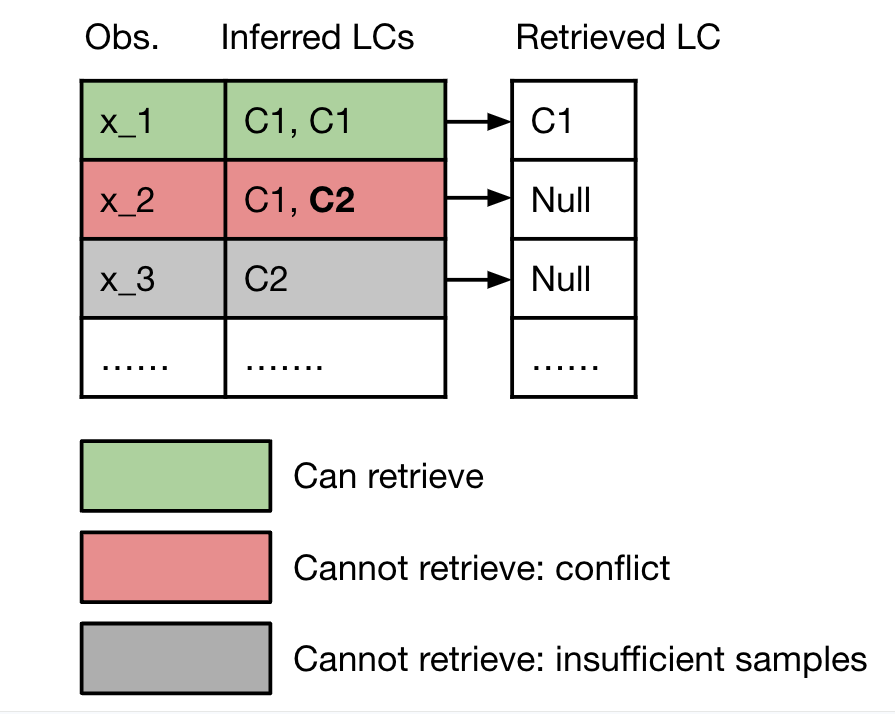


Figure S5

- An episodic memory buffer that keeps the most recent *M* (= 2) latent causes for each observation *x*. Given *x*_1_, it will retrieve C1 because it stored 2 latent causes associated with *x*_1_, and all of them are the same. Given *x*_2_, it will not retrieve anything because there is a conflict (i.e., C2) across stored memories. Given *x*_3_, it will not retrieve anything because it has not stored enough samples for this observation.

Finally, we need to specify when to use episodic memory versus full LCI (Algorithm 3). The rule is simple – given an observation *x*, if an episodic memory is retrieved, the model will use the retrieved LC and suppress the full inference procedure. Once episodic memory is activated for *x*, it will remain active for *x* until the model experiences a high loss for *x*, in which case episodic retrieval for *x* will be turned off, and the row corresponding to *x* in the episodic buffer will be cleared. The model tracks the running mean and standard deviation of the losses conditioned on the observations when full inference is active. The loss is considered to be too high (i.e., loss/prediction error peak) if it is higher than the mean plus three standard deviations, estimated from the history of losses for this observation.

##### Algorithm 3

**Handoff between episodic memory and full latent cause inference**

**Given** an observation *x_t_*

Retrieve episodic memory based on *x_t_*

**If** a latent cause, denoted by *C_m_*, is retrieved:

*x_t_*_+1 =_  model (*x_t_*_,_ *C_m_*)

**If** there is a prediction error (loss) peak:

clear the memory buffer for *x* (which will turn off episodic memory for *x*)

**else**:

*C_f_* = full_inference(*x_t_*)

memory_buffer.store(*x_t_*, *C_f_*)

*x_t_*_+1 =_  model (*x_t_*_,_ *C_f_*)

record the loss and update the mean and standard deviation of the loss

###

###

### Supplement 5

**Exploratory analysis of inferred latent causes**

**
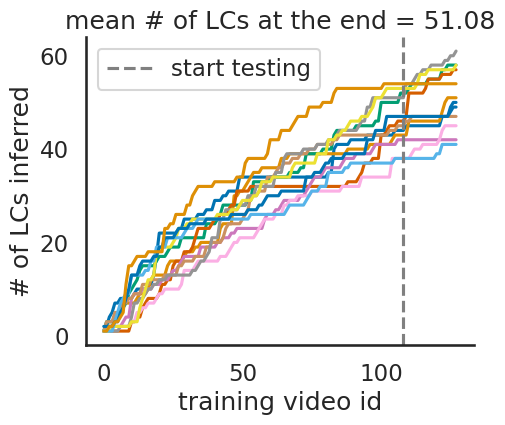
**

##### Figure S6

Number of inferred events over time. Each curve is one model. Overall, the number of inferred latent causes grows linearly as the model views more videos.


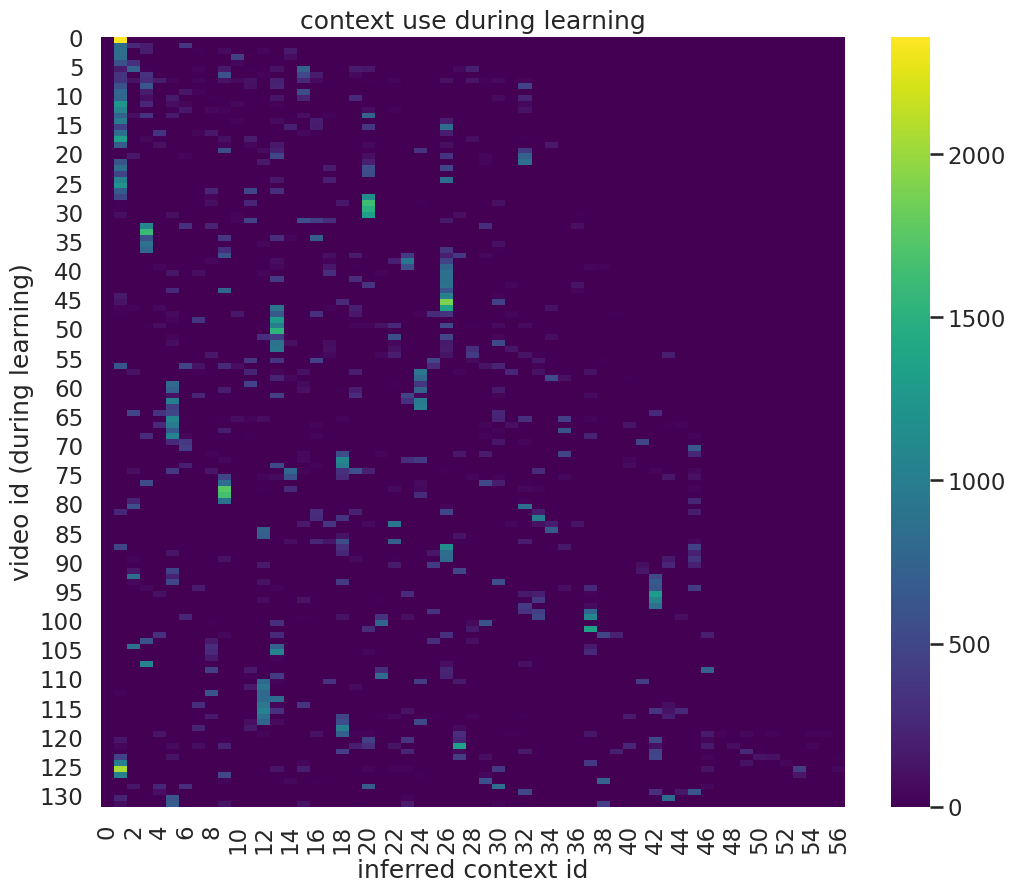


##### Figure S7

Evidence of reusing latent causes from one example. As LCNet was being trained on META, it reused previously generated latent causes. Here is a heatmap of the number of time points assigned to each of the latent causes for each training video for one example model. A row in this matrix tells us the distribution of latent causes within a video. A column in this matrix tells us how a latent cause was used throughout the training.

###

### Supplement 6

**A comparison between LCNet and SEM 2.0 on Simulation 3**

###
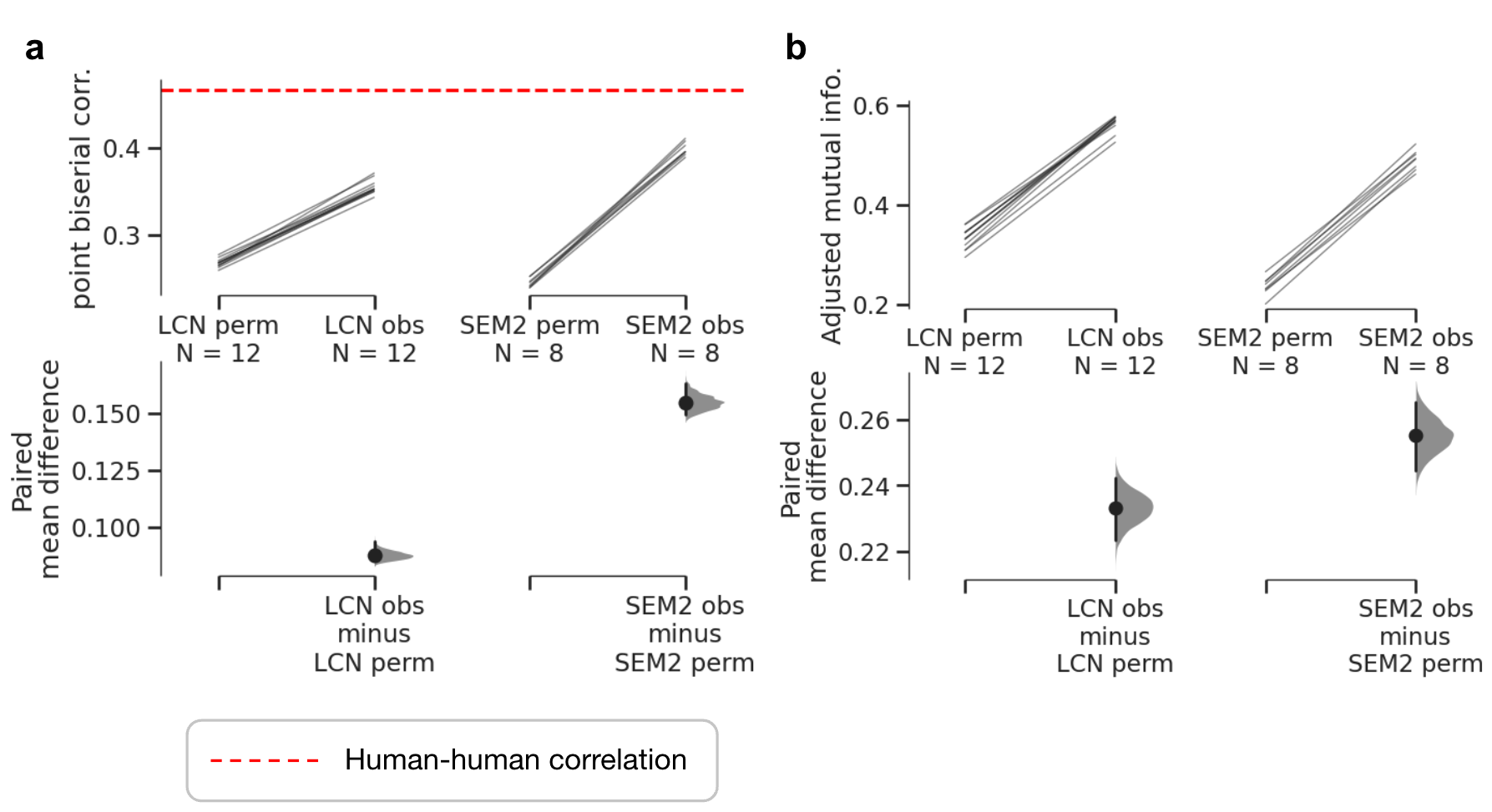


##### Figure S8

A comparison between LCNet vs. SEM 2.0 shows that LCNet is slightly worse compared to SEM 2.0 in terms of (a) point biserial correlation with human data and (b) adjusted mutual information with the ground truth low-level event labels. This is potentially due to several additional mechanisms in SEM 2.0, such as the fact that it tracks the variance of prediction error in an LC-specific manner, as well as other differences in the implementation details.

###

###

### Supplement 7

For each low-level event category (i.e., the leaves in the ground truth hierarchy shown in Figure 4a), we compute the mean activity pattern of the recurrent hidden layer. Then we compute the representational dissimilarity matrix (RDM)[^14,15^](https://paperpile.com/c/c9vAlV/Q1gtP+QP1QQ) over low-level event categories (Figure 4i). The task RDM is computed using the scene vectors (Figure 4h). The result shows that the correlation between the model RDM and the task RDM is significant (Figure 4j; p < 1e-5).


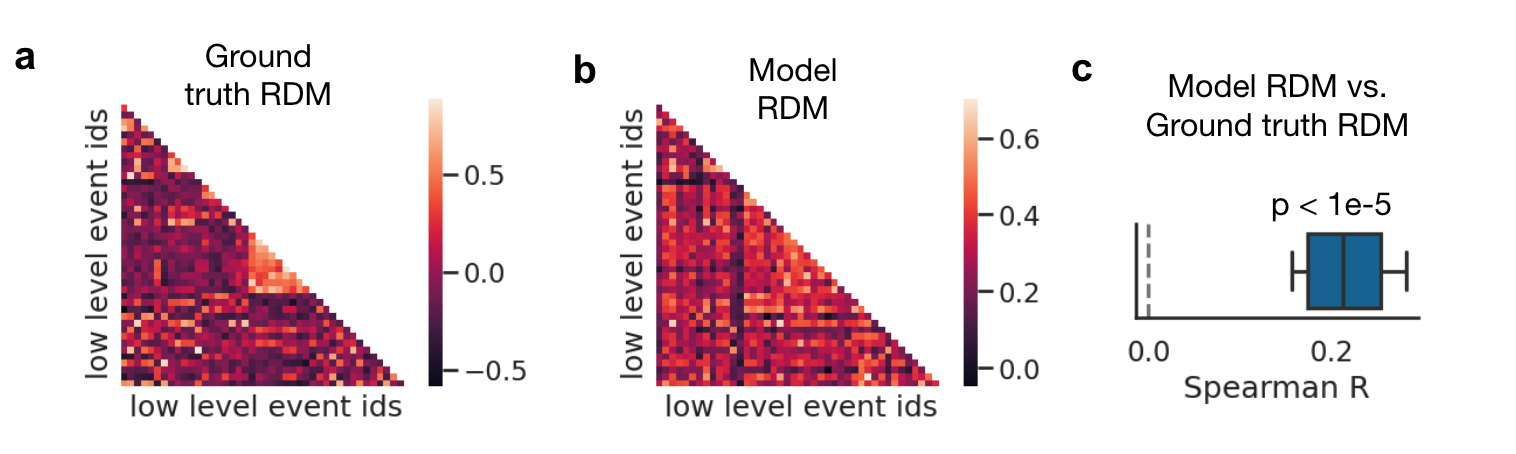


##### Figure S9

a) The representational dissimilarity matrix (RDM) for the mean patterns averaged within each low-level event.

b) The RDM for the LCNet hidden state averaged within each low-level event.

c) The correlation between the model RDM and the ground truth RDM.

### Supplement 8

**Hyperparameters**

We implemented the model in PyTorch[^16,17^](https://paperpile.com/c/c9vAlV/3RHPj+OsO21). In Simulation 1, the number of hidden units and learning rate were equated across models for model comparison. Minor changes in these parameters did not influence the general pattern that i) LCNet suffers less catastrophic interference compared to a regular neural network and ii) LCNet learns new tasks faster than SEM (see Supplement 9).

In Simulation 2, the goal is to show that there is a setting where a blocked curriculum allowed the model to perform better during the test phase, compared to an interleaved curriculum. Therefore, we optimized stickiness and concentration by a hyper-parameter grid search, and used parameters that maximized the model performance in the blocked curriculum. Additionally, we showed that, when using a lower stickiness value, LCNet can perform better in the interleaved condition (Supplement 3). The number of hidden units and learning rate were chosen so that the model did not require too many trials in the experiment to learn the tasks. Minor changes in the number of hidden units and learning rate did not influence the main finding regarding the impact of stickiness on the relative performance between blocked and interleaved conditions.

In Simulation 3, similar to how these parameters were chosen in SEM 2.0[^18^](https://paperpile.com/c/c9vAlV/2P9fo), the stickiness and concentration parameters were chosen to ensure the number of event boundaries, mean event duration, and average total number of latent causes of the model qualitatively matched with human data. The number of hidden units and the optimizer were also matched with SEM 2.0 to facilitate model comparison. Finally, in all three simulations, we set the dimension of the context vectors to 128 to ensure sufficient orthogonality without adding too many parameters to the model (Supplement 1).

We used the following hyperparameters for the three simulations:

Simulation 1:

- Optimizer: Adam[^19^](https://paperpile.com/c/c9vAlV/G0w2a)
- Learning rate: 1 x 10^-5^
- Number of hidden units: 128
- The dimension of the context vectors: 128
- The dimension of the context-indicative signal: 128
- Nonlinearity for hidden units: ReLU

Simulation 2:

- Optimizer: Adam[^19^](https://paperpile.com/c/c9vAlV/G0w2a)
- Learning rate: 4 x 10^-3^
- Number of hidden units: 128
- *w*, the weight on the current state when computing the running average of the input: 0.8
- The dimension of the context vectors: 128
- sCRP prior
  - Stickiness: 32
- Concentration: 0.5
- Nonlinearity for hidden units: Sigmoidal

Simulation 3:

- Optimizer: Adam[^19^](https://paperpile.com/c/c9vAlV/G0w2a)
- Learning rate: 1 x 10^-3^
- Number of hidden units: 16
- The dimension of the context vectors: 128
- sUP prior
  - Stickiness: 4
  - Concentration: 1
- Nonlinearity for hidden units: we used the standard nonlinearity in Gated Recurrent Units (GRU) specified in the GRU methods section.

###

###

### Supplement 9

**Additional manipulation of hyperparameters in Simulation 1**

Here, we conduct additional experiments to ensure that minor changes of the two hyperparameters, the number of hidden units and learning rate, do not influence the main findings that i) LCNet suffers less catastrophic interference compared to a regular neural network and ii) LCNet learns new tasks faster than SEM. In general, decreasing/increasing the number of hidden units or learning rate decreases/increases the learning efficiency for all three models.

**
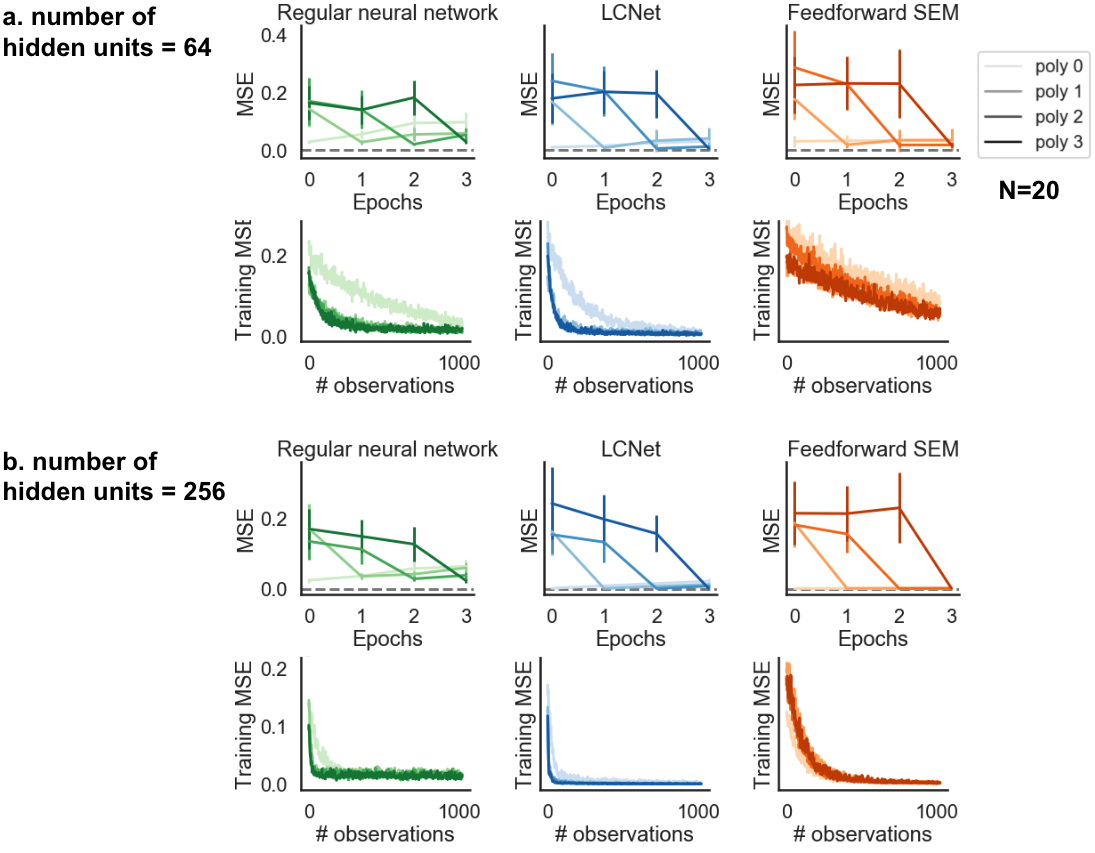
**

##### Figure S10

Here, we report the main analyses (see Figure 2h, 2i) when the number of hidden units (which is 128 in the main paper) is changed to 64 (a) and 256 (b). The same pattern reported in the main paper qualitatively holds. Error bars indicate 3SE. *N* = 20 models per condition.


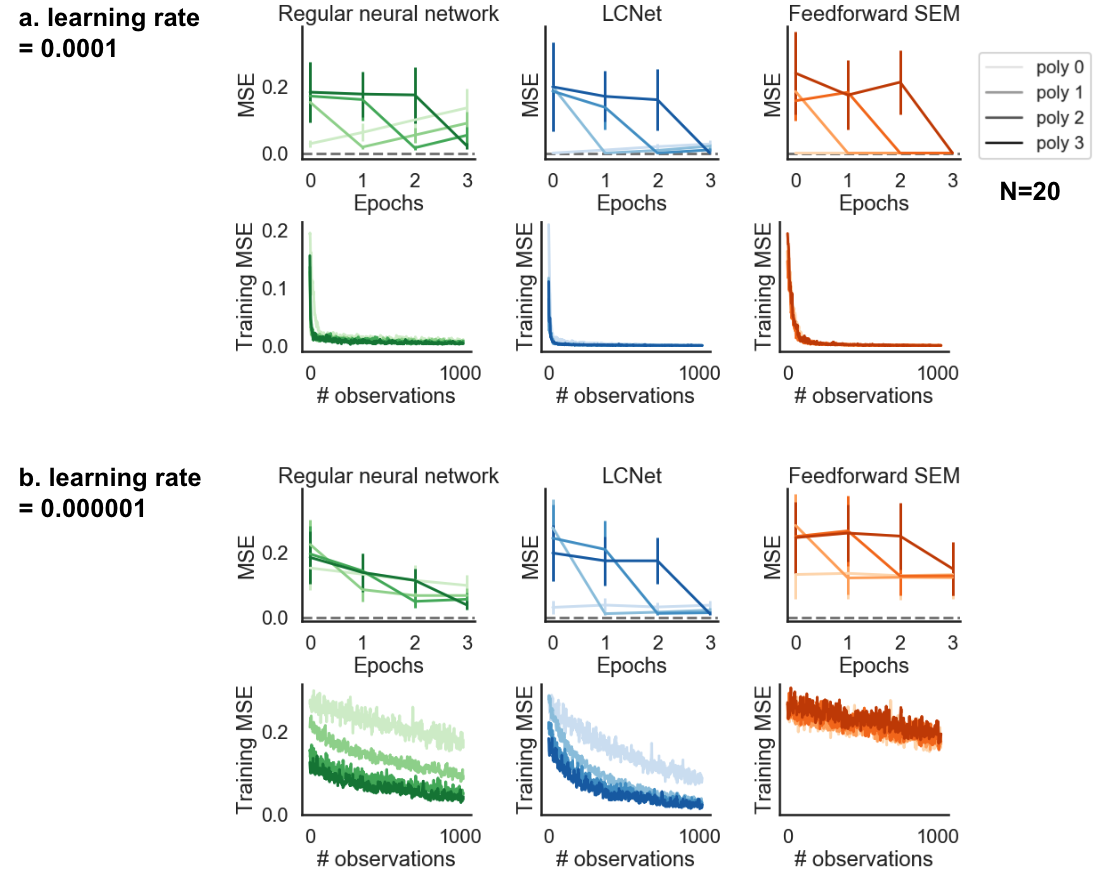


##### Figure S11

Here, we report the main analyses (see Figure 2h, 2i) when the learning rate (which is 1 x 10^-5^ in the main paper) is changed to 1 x 10^-4^ (a) and 1 x 10^-6^ (b). The same pattern reported in the main paper qualitatively holds. Error bars indicate 3SE. *N* = 20 models per condition.

#

## References:

1. [Dasgupta, I., Schulz, E., Goodman, N. D. & Gershman, S. J. Remembrance of inferences past: Amortization in human hypothesis generation. *Cognition* **178**, 67–81 (2018).](http://paperpile.com/b/c9vAlV/6PmPY)

2. [Ritter, S. *et al.* Been There, Done That: Meta-Learning with Episodic Recall. in *Proceedings of the International Conference on Machine Learning (ICML).* (2018).](http://paperpile.com/b/c9vAlV/TQUGn)

3. [Lu, Q., Hasson, U. & Norman, K. A. A neural network model of when to retrieve and encode episodic memories. *Elife* **11**, e74445 (2022).](http://paperpile.com/b/c9vAlV/LwTd2)

4. [Pritzel, A. *et al.* Neural Episodic Control. *arXiv [cs.LG]* (2017).](http://paperpile.com/b/c9vAlV/DYKRk)

5. [Richards, B. A. & Frankland, P. W. The Persistence and Transience of Memory. *Neuron* **94**, 1071–1084 (2017).](http://paperpile.com/b/c9vAlV/4UPRi)

6. [Yalnizyan-Carson, A. & Richards, B. A. Forgetting Enhances Episodic Control With Structured Memories. *Front. Comput. Neurosci.* **16**, 757244 (2022).](http://paperpile.com/b/c9vAlV/m9GMm)

7. [McClelland, J. L., McNaughton, B. L. & O’Reilly, R. C. Why there are complementary learning systems in the hippocampus and neocortex: insights from the successes and failures of connectionist models of learning and memory. *Psychol. Rev.* **102**, 419–457 (1995).](http://paperpile.com/b/c9vAlV/D8dFG)

8. [McClelland, J. L. Incorporating rapid neocortical learning of new schema-consistent information into complementary learning systems theory. *J. Exp. Psychol. Gen.* **142**, 1190–1210 (2013).](http://paperpile.com/b/c9vAlV/Oq6ly)

9. [Norman, K. A. & O’Reilly, R. C. Modeling hippocampal and neocortical contributions to recognition memory: a complementary-learning-systems approach. *Psychol. Rev.* **110**, 611–646 (2003).](http://paperpile.com/b/c9vAlV/DWzYT)

10. [O’Reilly, R. C., Bhattacharyya, R., Howard, M. D. & Ketz, N. Complementary learning systems. *Cogn. Sci.* **38**, 1229–1248 (2014).](http://paperpile.com/b/c9vAlV/DtcZy)

11. [Norman, K. A., Detre, G. & Polyn, S. M. Computational models of episodic memory. in *The Cambridge handbook of computational psychology , (pp* (ed. Sun, R.) vol. 753 189–225 (Cambridge University Press, xii, New York, NY, US, 2008).](http://paperpile.com/b/c9vAlV/ofWCP)

12. [Norman, K. A. How hippocampus and cortex contribute to recognition memory: revisiting the complementary learning systems model. *Hippocampus* **20**, 1217–1227 (2010).](http://paperpile.com/b/c9vAlV/CrTZe)

13. [O’Reilly, R. C. & Norman, K. A. Hippocampal and neocortical contributions to memory: advances in the complementary learning systems framework. *Trends Cogn. Sci.* **6**, 505–510 (2002).](http://paperpile.com/b/c9vAlV/aZRDx)

14. [Kriegeskorte, N., Mur, M. & Bandettini, P. Representational similarity analysis - connecting the branches of systems neuroscience. *Front. Syst. Neurosci.* **2**, 4 (2008).](http://paperpile.com/b/c9vAlV/Q1gtP)

15. [Kriegeskorte, N. *et al.* Matching categorical object representations in inferior temporal cortex of man and monkey. *Neuron* **60**, 1126–1141 (2008).](http://paperpile.com/b/c9vAlV/QP1QQ)

16. [Paszke, A. *et al.* Automatic differentiation in PyTorch. (2017).](http://paperpile.com/b/c9vAlV/3RHPj)

17. [Paszke, A. *et al.* PyTorch: An Imperative Style, High-Performance Deep Learning Library. *arXiv [cs.LG]* (2019).](http://paperpile.com/b/c9vAlV/OsO21)

18. [Bezdek, M. A. *et al.* Uncertainty-driven updating enables human-like segmentation and categorization of naturalistic activity. *PsyArXiv* (2022) doi:](http://paperpile.com/b/c9vAlV/2P9fo)[10.31234/osf.io/pt6hx](http://dx.doi.org/10.31234/osf.io/pt6hx)[.](http://paperpile.com/b/c9vAlV/2P9fo)

19. [Kingma, D. P. & Ba, J. Adam: A Method for Stochastic Optimization. *arXiv [cs.LG]* (2014).](http://paperpile.com/b/c9vAlV/G0w2a)

20. [De Soares, A. *et al.* Top-down attention shifts behavioral and neural event boundaries in narratives with overlapping event scripts. *bioRxiv* 2023.08.08.552465 (2023) doi:](http://paperpile.com/b/c9vAlV/W0pB)[10.1101/2023.08.08.552465](http://dx.doi.org/10.1101/2023.08.08.552465)[.](http://paperpile.com/b/c9vAlV/W0pB)

21. [Wang, J. X. *et al.* Prefrontal cortex as a meta-reinforcement learning system. *Nat. Neurosci.* **21**, 860–868 (2018).](http://paperpile.com/b/c9vAlV/ierpf)
